# Platelet/Albumin ratio and plateletcrit levels are potential new biomarkers for assessing endoscopic inflammatory bowel disease severity

**DOI:** 10.1186/s12876-023-03043-4

**Published:** 2023-11-14

**Authors:** Jun Huang, Jie Lu, Feiyu Jiang, Tiejun Song

**Affiliations:** 1 Clinical Laboratory, Sir Run Run Shaw Hospital, Zhejiang University School of Medicine, Hangzhou, China; 2 Key Laboratory of Precision Medicine in Diagnosis and Monitoring Research of Zhejiang Province, Hangzhou, China

**Keywords:** Inflammatory bowel Disease, Endoscopic Disease activity, Platelet, Plateletcrit, Platelet-to-albumin ratio

## Abstract

**Background:**

Endoscopy is currently recognized as the gold standard for assessing inflammatory bowel disease (IBD) severity. However, because the procedure is costly and invasive, endoscopy is not suitable for frequently monitoring intestinal inflammation. In this study, our aim was to identify noninvasive, low cost, and convenient biomarkers for identifying endoscopic IBD activity.

**Methods:**

In total, 246 patients with IBD (131 with Ulcerative colitis (UC) and 115 with Crohn’s disease (CD)) and 369 healthy controls were recruited for this retrospective study. IBD activity was evaluated using endoscopic and clinical examinations. The potential of several inflammatory biomarkers, including platelets (PLT), plateletcrit (PCT), albumin (ALB), highly sensitive C-reactive protein (hs-CRP), erythrocyte sedimentation rate (ESR), and platelet-to-albumin ratio (PLT/ALB) to assess endoscopic IBD activity was evaluated using receiver operating characteristic (ROC) analyses.

**Results:**

PLT/ALB ratio, PLT, ALB, and hs-CRP levels were correlated with Mayo scores in UC patients, while PCT, PLT, fibrinogen (FIB), PLT/ALB ratio, hs-CRP, and ESR levels were correlated with Simple Endoscopic Scores for CD (SES-CD) in CD patients. ROC analyses showed that the area under the curve (AUC) value for the PLT/ALB ratio (0.705) was greater than hs-CRP (0.607) and ESR (0.552) values in UC patients. The AUC value for PCT (0.779) was greater than hs-CRP (0.698) and ESR (0.746) values in CD patients.

**Conclusion:**

PLT/ALB ratio and PCT biomarkers were the most appropriate of all tested inflammatory biomarkers for assessing endoscopic IBD activity in UC and CD patients, respectively.

## Background

Inflammatory bowel disease (IBD), including ulcerative colitis (UC) and Crohn’s disease (CD), is characterized by recurrent or persistent inflammatory disorders of the gastrointestinal tract which may cause digestive disability [1] and result from genetic [2], environmental [3], and immunological factors [4]. IBD symptoms include diarrhea, abdominal pain, and bloody stools. Frequent disease activity monitoring is crucial for patients with IBD, as it ensures prompt therapeutic strategies and improves prognosis and quality of life [5].

Endoscopy is widely considered the gold standard for diagnosing UC and CD, prognosticating disease severity, and more recently, evaluating mucosal response to therapy [6]. Several endoscopy scoring systems have been established to classify IBD activity, including Mayo endoscopic subscores for UC and Simple Endoscopic Score for CD (SES-CD) [7, 8]. Although costly and limited by its invasiveness, endoscopy remains the most reliable approach for monitoring patient progress over time [9]. Non-endoscopic indices, including the Simple Clinical Colitis Activity Index (SCCAI) for UC and the Harvey Bradshaw Index (HBI) for CD, are responsive scores with clear definitions indicating clinical responses and remission information [7]. However, they do not correlate well with intestinal inflammation [10]. Therefore, ideal noninvasive markers must be identified to reflect endoscopic IBD activity.

Different studies have suggested that some noninvasive and low cost biomarkers can accurately monitor IBD activity. Faecal calprotectin (FCP) is a reliable biomarker for evaluating endoscopic disease activity in IBD. However, FCP has not routinely been used in some countries and regions. Some patients may forget or be reluctant to perform this test [11]. High sensitive C-reactive protein (hs-CRP) and erythrocyte sedimentation rate (ESR) levels are commonly used to assess clinical disease activity in IBD, however, their roles determining mucosal inflammation levels remain controversial [12–14]. A recent study reported that fibrinogen (FIB) was a valuable biomarker identifying active stage IBD [15]. Platelets (PLT) have essential roles in UC and CD pathogenesis and may be better predictive markers for endoscopic IBD activity when compared with CRP and ESR [16, 17]. For serum albumin (ALB), apart from its traditional role in nutrition, it is widely recognized as a negative acute phase protein, with ALB levels directly affected by acute infection severity [18]. A recent study reported that ALB levels had high sensitivity and specificity for potential CD [19].

In this study, we investigated associations between FIB, PLT, plateletcrit (PCT), ALB, and a serological optimizing marker, platelet-to-albumin ratio (PLT/ALB) and endoscopic and clinical scores, and validated their diagnostic value in identifying “low” or “high” IBD activity when compared with endoscopic examinations.

## Materials and methods

### Patients

From January 2017 to October 2018, 246 patients with IBD (n = 131 UC patients and n = 115 CD patients) were recruited to this retrospective study at Sir Run Run Shaw Hospital, Zhejiang University School of Medicine, Hangzhou, China. Patients were diagnosed with IBD based on standard clinical, laboratory, radiological, endoscopic, and histopathological findings. Age, gender, disease course, clinical symptom, ileocolonoscopic, and radiographic examination data were collected using electronic medical records. In terms of healthy controls (HCs), 369 were selected based on age, sex, and registration year to ensure similar healthcare exposure between patients with IBD and HCs. HCs underwent general physical examinations within 1 year of their corresponding case diagnosis, with normal findings identified. HCs were subjected to the same inclusion and exclusion criteria as patients with IBD.

Exclusion criteria were pregnancy, concomitant inflammatory disorders, peripheral vascular disease, cancer, renal insufficiency, liver injury, and diabetes. No participants received anticoagulant medications or contraceptives. The need for written informed consent to participate was waived by the Sir Run Run Shaw Hospital, Zhejiang University School of Medicine ethics committee due to retrospective nature of the study.

### Biochemical and hematological analyses

The timing of endoscopic examinations in IBD patients ranged from half a month to one year after the identification of symptoms. Blood samples were collected within three months prior to the endoscopic examination. Samples were obtained from the antecubital vein in ethylene diamine tetra-acetic acid vacutainer tubes (BD, NJ, USA) for the hematological analysis, serum separation tubes (BD, NJ, USA) for the biochemical analysis and citrated tubes (BD, NJ, USA) for the coagulation analysis, mixing one part 3.2% trisodium citrate and nine parts blood. All blood samples were transported to a clinical laboratory at Sir Run Run Shaw Hospital, and analyses were performed on fresh samples.

Serum hs-CRP and ALB values were quantified using a C-16,000 plus biochemistry analyzer (Abbott, Tochigi, Japan). According to standard protocols, ESR was measured using a MONTIOR 100 auto ESR analyzer (VITAL, Forli, Italy).

White blood cells (WBCs), hemoglobin (Hb), absolute values of different leukocytes (neutrophils, lymphocytes, monocytes, eosinophils, and basophils), PLT counts, and PCT were quantified in whole blood samples using the Coulter 780 5 Diff analyzer (Beckman Coulter, CA, USA). PLT/ALB, platelet-to-lymphocyte (PLR), and neutrophil-to-lymphocyte ratios (NLR) were calculated based on neutrophil, lymphocyte, PLT, and ALB levels. FIB levels were assessed in plasma samples using STA-R Evolution instrumentation (Stago, Asnieres-sur-Seine, France).

### Defining endoscopic IBD activity

Endoscopic disease activity was graded based on available endoscopic images and endoscopy reports written by certified gastroenterologists from our hospital. Endoscopic IBD activity was classified according to the Mayo scoring system for UC, and the Simplified Endoscopic Scores for CD (SES-CD). A Mayo 0 score was defined as endoscopic remission, Mayo 1 as mild UC activity, Mayo 2 as moderate UC activity, and Mayo 3 as severe UC activity. SES-CD 0–3 points indicated remission, 4–10 points indicated mild CD activity, 11–19 points indicated moderate CD activity, and ≥ 20 points indicated severe CD activity [20, 21]. For analyses, categories from both endoscopy indices; SES-CD and Mayo endoscopic UC subscores were merged into two groups: patients with low endoscopic disease activity (Mayo 0 or 1 in UC and SES-CD < 10 in CD) and patients with high endoscopic disease activity (Mayo 2 or 3 in UC and SES-CD ≥ 10 in CD).

### The clinical status of patients with IBD

The SCCAI and HBI are reliable and responsive scores which provide clear definitions for clinical responses and remission in UC and CD, respectively. SCCAI scores range between 0 and 19 points. An SCCAI score of < 2 indicated clinical remission in UC. A HBI score of < 5 indicated clinical remission in CD, 5–7 points as mild CD activity, 8–16 points as moderate CD activity, and > 16 as severe CD activity [7].

### Statistical analyses

The Statistical Package for the Social Sciences (SPSS Inc., Chicago, IL, USA, Version 19.0) was used for statistical analyses. GraphPad Prism 5 (GraphPad, San Diego, CA, USA) was used to generate plots. Normal variables were determined using Kolmogorov-Smirnov tests. Normally distributed variables are expressed as the mean ± SD. Non-normally distributed variables were expressed as the medians with interquartile ranges (IQRs). To compare > two groups, one-way analysis of variance was used for parametric variables and Kruskal-Wallis tests for nonparametric variables. Spearman’s correlation analyses were used to explore relationships between parameters and Mayo or SES-CD scores. The ability of PLT/ALB ratios and other variables to differentiate between patients with mild and severe endoscopic disease activity was evaluated using receiver operating characteristic (ROC) curves. Statistical significance was set at p < 0.05. Cut-off points were estimated using the best combinations according to Youden’s J statistic (J = Sensitivity + Specificity − 1). Area under the ROC curve (AUC) was used to obtain another estimate of the diagnostic accuracy of these biomarkers.

## Results

### Patient characteristics

The demographic and clinical characteristics of patients with IBD and HCs are shown (Table 1). In total, 246 patients with IBD, including 131 UC and 115 CD patients, and 369 HCs were enrolled. The median ages of UC and CD patients, and HCs were 46 and 49, and 47 years, respectively. The age of the patients is referring to the time of sampling. From Mayo scores in the UC group, 16.1% of patients had low UC activity (remission to mild UC activity) and 83.9% had moderate to severe UC activity. From SES-CD scores in the CD group, 70.5% patients had low CD activity (remission to mild CD activity) and 29.5% had moderate to severe CD activity.

**Table 1:** Patient characteristics

Characteristics	UC (n = 131)	CD (n = 115)	HCs (n = 369)
**Age (years)**	46 (30, 56)	49 (33, 57)	47 (32, 67)
**Gender; male (female)**	73 (58)	76 (39)	237 (132)
**Endoscopic activity**			
Remission	6 (4.6%)	37 (32.2%)	-
Mild	15 (11.5%)	44 (38.3%)	-
Moderate	54 (41.2%)	25 (21.7%)	-
Severe	56 (42.7%)	9 (7.8%)	-
**Lesion location**			-
UC Proctis	9 (6.9%)	-	-
Distal colitis	22 (16.7%)		
Left sided	60 (45.8%)	-	-
Extensive	9 (6.9%)		
Pancolitis	31 (23.7%)	-	-
CD Ileitis	-	22 (19.1%)	-
Colitis	-	7 (6.1%)	-
Ileocolitis	-	76 (66.1%)	-
Perianal lesions	-	10 (8.7%)	-
**Avoided surgery**			
Yes	97 (74.0%)	101 (87.8%)	-
No	34 (26.0%)	14 (12.2%)	-

**Abbreviations**: UC, ulcerative colitis; CD, Crohn’s disease; HCs, healthy controls

### Biomarker analyses in HCs and patients with IBD

Ten inflammatory biomarkers and their concentrations in IBD patients and HCs are shown (Table 2). Patients with UC and CD had higher median hs-CRP, ESR, FIB, PLT, PCT, NLR, PLR, and PLT/ALB ratio values, and lower median Hb and ALB values when compared with HCs (p < 0.001). Furthermore, PLT, FIB, PLR, and PLT/ALB ratio levels were significantly higher in CD when compared with UC patients (p < 0.001).

**Table 2:** Clinical parameters in patients with UC and CD, and HCs.

Parameters	UC (n = 131)	CD (n = 115)	HCs (n = 369)	p
**hs-CRP (mg/L)**	8.20 (2.20, 25.45)^a^	14.80 (6.10, 40.20)^b^	0.90 (0.50, 2.20)	< 0.001
**ESR (mm/hr)**	18.00 (10.00, 33.50)^a^	17.00 (7.00, 36.00)^b^	7.00 (4.00, 11.00)	< 0.001
**Hb (g/L)**	12.00 (10.20, 13.40)^a^	11.90 (10.40, 13.00)^b^	13.70 (12.90, 14.70)	< 0.001
**FIB (g/L)**	3.81 (3.25, 4.87)^a,c^	4.63 (3.37, 5.50)^b^	2.71 (2.39, 3.03)	< 0.001
**PLT × 10** ^**9**^ **/L**	265.00 (211.00, 324.00)^a,c^	305.00 (236.00, 394.00)^b^	201.50 (167.75, 224.75)	< 0.001
**PCT (%)**	0.22 (0.18, 0.25)^a^	0.24 (0.20, 0.30)^b^	0.19 (0.16, 0.20)	< 0.001
**ALB (g/L)**	35.10 (30.40, 38.90)^a^	36.20 (33.10, 40.40)^b^	43.65 (41.50, 44.80)	< 0.001
**NLR**	3.18 (2.04, 5.59)^a^	3.17 (2.21, 4.52)^b^	1.86 (1.39, 2.44)	< 0.001
**PLR**	176.07 (121.60, 301.55)^a,c^	246.78 (177.09, 364.32)^b^	114.37 (91.55, 144.03)	< 0.001
**PLT/ALB**	7.76 (5.53, 10.06)^a,c^	8.64 (6.60, 11.29)^b^	4.86 (3.57, 5.25)	< 0.001

^a^UC group vs. HC group, p < 0.05; ^b^CD group vs. HC group, p < 0.05. ^c^UC group vs. CD group, p < 0.05. Data are presented as the median (interquartile range (IQR)). Differences between groups were tested using one-way analysis of variance; p < 0.05 was considered statistically significant

**Abbreviations**: UC, ulcerative colitis; CD, Crohn’s disease; HCs, healthy controls, hs-CRP, high sensitive C-reactive protein; ESR, erythrocyte sedimentation rate; Hb, hemoglobin; FIB, fibrinogen; PLT, platelet; PCT, plateletcrit; ALB, Albumin; NLR, neutrophil-to-lymphocyte ratio; PLR, platelet-to-lymphocyte ratio; PLT/ALB, platelet-to-albumin ratio

### Inflammatory biomarker correlations with endoscopic IBD activity

To analyze correlations between inflammatory biomarkers and clinical (SCCAI for UC and HBI for CD) and endoscopic (Mayo scores for UC and SES-CD scores for CD) measures of disease activity **(**Table 3), we used Spearman’s correlation analyses. Mayo scores were significantly positively correlated with only three biomarkers: hs-CRP (r = 0.198, p = 0.026), PLT (r = 0.231, p = 0.009), and PLT/ ALB (r = 0.293, p = 0.001), and negatively correlated with ALB (r = − 0.298, p = 0.001) in UC patients. SES-CD scores were significantly positivity correlated with hs-CRP (r = 0.313, p = 0.001), ESR (r = 0.298, p = 0.001), WBC (r = 0.258, p = 0.005), FIB (r = 0.234, p = 0.013), PCT (r = 0.357, p < 0.001), PLT (r = 0.303, p = 0.001), and PLT/ALB (r = 0.284, p = 0.002) in CD patients. However, HBI only showed significant correlations with ESR (r = 0.241, p = 0.010), PLT/ALB (r = 0.195, P = 0.037), and ALB (r = − 0.291, p = 0.002).

**Table 3:** Correlations between inflammatory biomarkers and endoscopic IBD activity and clinical measures

	UC			CD	
	Mayo	SCCAI		SES-CD	HBI
**hs-CRP (mg/L)**	0.198*	0.183*		0.313**	0.18
**ESR (mm/hr)**	0.169	0.135		0.298**	0.241**
**WBC × 10** ^**9**^ **/L**	0.104	0.206*		0.258**	0.115
**FIB (g/L)**	0.099	0.219*		0.234**	0.132
**PCT (%)**	0.035	0.049		0.357**	0.097
**PLT × 10** ^**9**^ **/L**	0.231**	0.280**		0.303**	0.102
**ALB (g/L)**	−0.298**	−0.262**		−0.092	−0.291**
**NLR**	0.075	−0.128		0.122	0.135
**PLR**	0.036	0.012		0.115	0.081
**PLT/ALB**	0.293**	0.277**		0.284**	0.195*

*p < 0.05, **p < 0.01, and p < 0.05 values were considered statistically significant (**bold**)

Endoscopic IBD activity and clinical measures: Mayo scores for UC and SES-CD for CD and SCCAI for UC and HBI for CD

**Abbreviations**: SCCAI, Simple Clinical Colitis Activity Index; SES-CD,Simplified Endoscopic Scores for CD; HBI, Harvey Bradshaw Index; hs-CRP, high sensitive C-reactive protein; ESR, erythrocyte sedimentation rate; WBC, white blood cell; FIB, fibrinogen; PLT, platelet; PCT, plateletcrit; ALB, Albumin; NLR, neutrophil-to-lymphocyte ratio; PLR, platelet-to-lymphocyte ratio; PLT/ ALB, platelet-to-albumin ratio

### Endoscopic disease activity assessment using inflammatory biomarkers

**Fig. 1:**
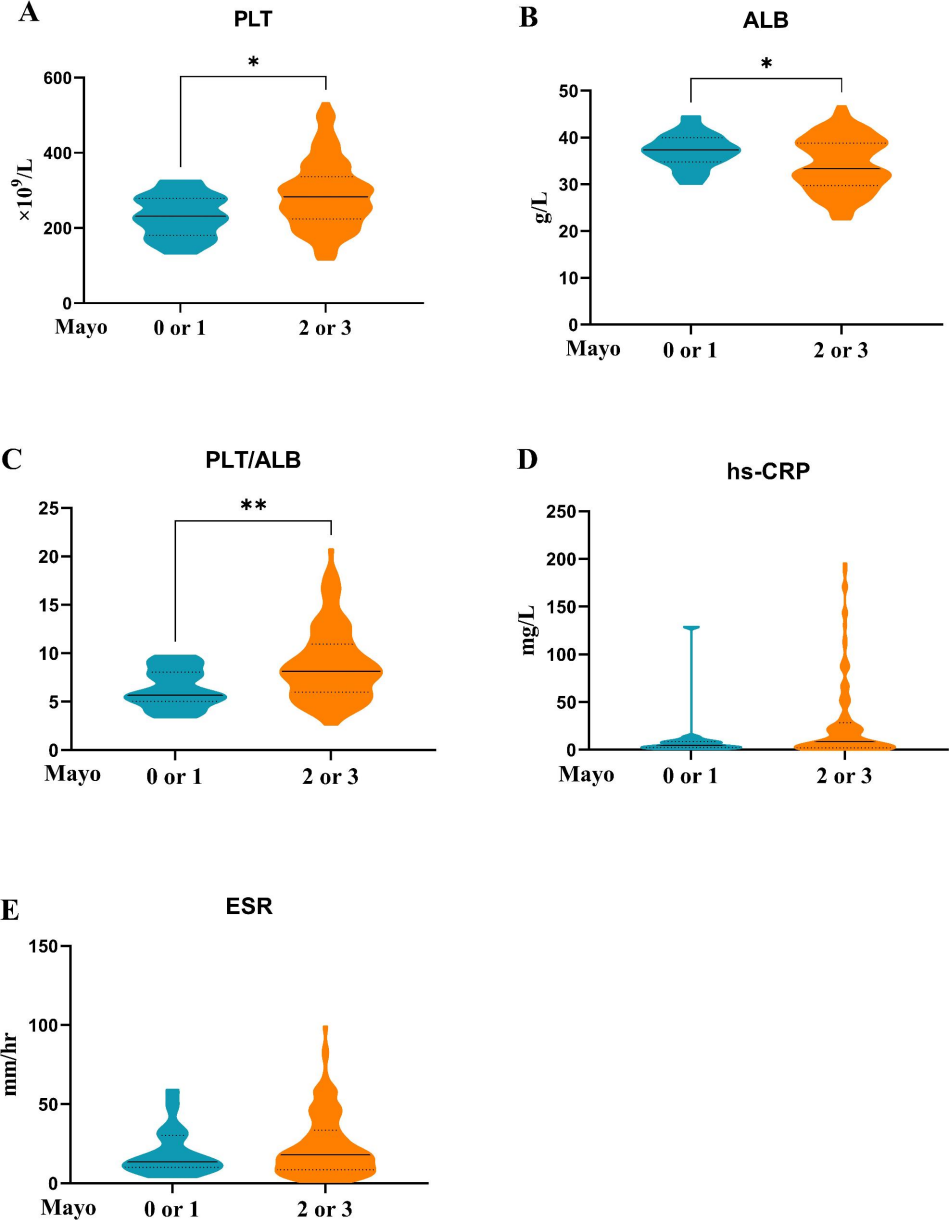
(**A**) platelet (PLT), (**B**) Albumin (ALB), (**C**) platelet-to-albumin ratio (PLT/ALB), (**D**) high sensitive C-reactive protein (hs-CRP), and (**E**) erythrocyte sedimentation rate (ESR) serum concentration differences between low endoscopic ulcerative colitis (UC) activity (Mayo 0 or 1) and high endoscopic UC activity (Mayo 1 or 2) in patients with UC.

**Fig. 2:**
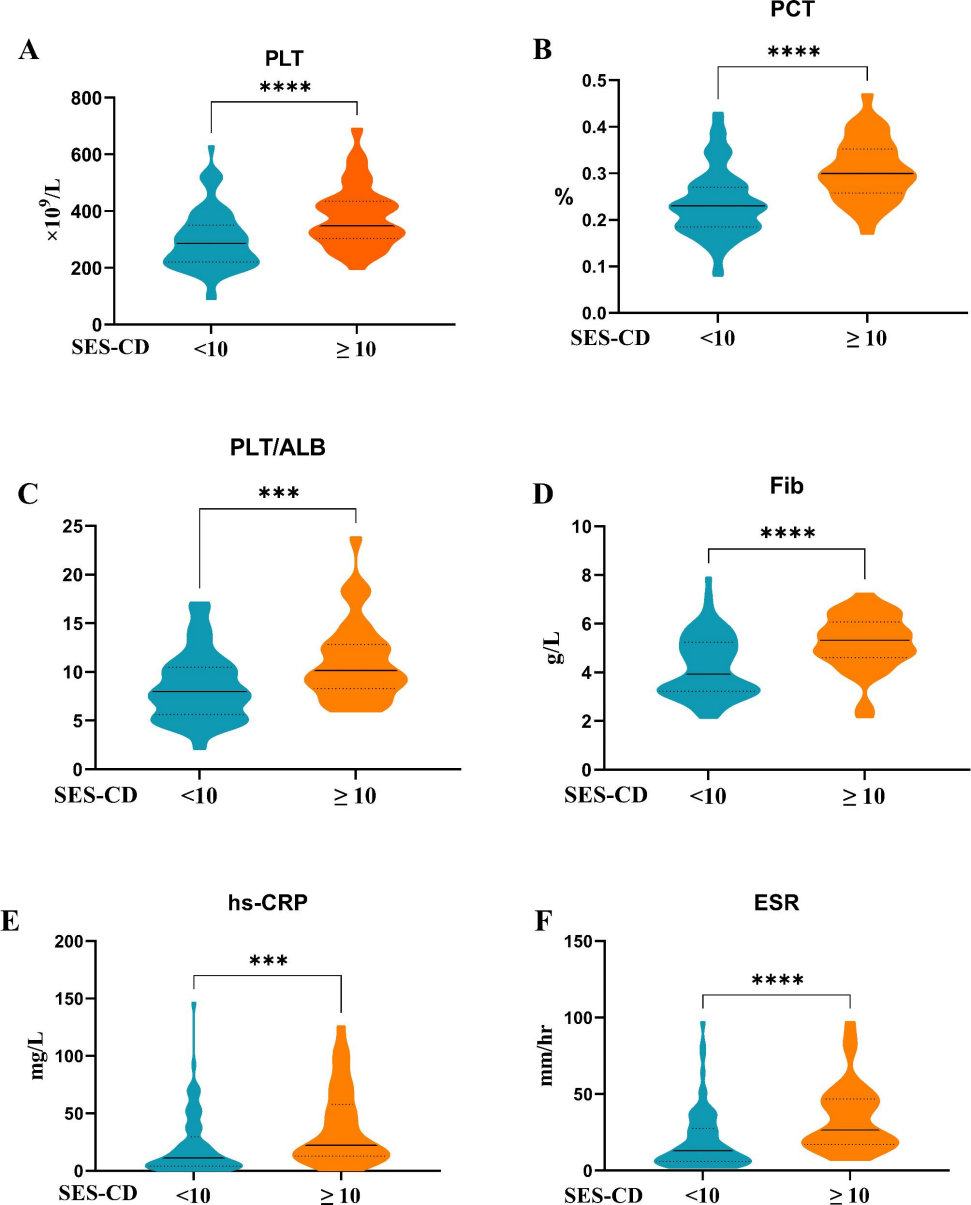
(**A**) platelet (PLT), (**B**) plateletcrit (PCT), (**C**) platelet-to-albumin ratio (PLT/ALB), (**D**) fibrinogen (FIB), (**E**) high sensitive C-reactive protein (hs-CRP), and (**F**) erythrocyte sedimentation rate (ESR) serum concentration differences between low endoscopic Crohn’s disease (CD) activity (SES-CD < 10) and high endoscopic CD activity (SES-CD ≥ 10) in patients with CD.

To assess diagnostic values for endoscopic disease activity, biomarker concentrations were compared between patients with low (remission and mild) and high endoscopic disease activity (moderate and severe) in UC (Fig. 1) and CD (Fig. 2).

From Mayo scores, UC patients with high endoscopic UC activity (Mayo 2 or 3) had significantly increased PLT and PLT/ALB levels and decreased ALB levels when compared with patients with low endoscopic UC activity (Mayo 0 or 1). From SES-CD scores, significantly increased PLT, PCT, PLT/ALB, FIB, hs-CRP, and ESR concentrations were observed in CD patients with high endoscopic CD activity (SES-CD ≥ 10) when compared with patients with low endoscopic CD activity (SES-CD < 10).

**Fig. 3:**
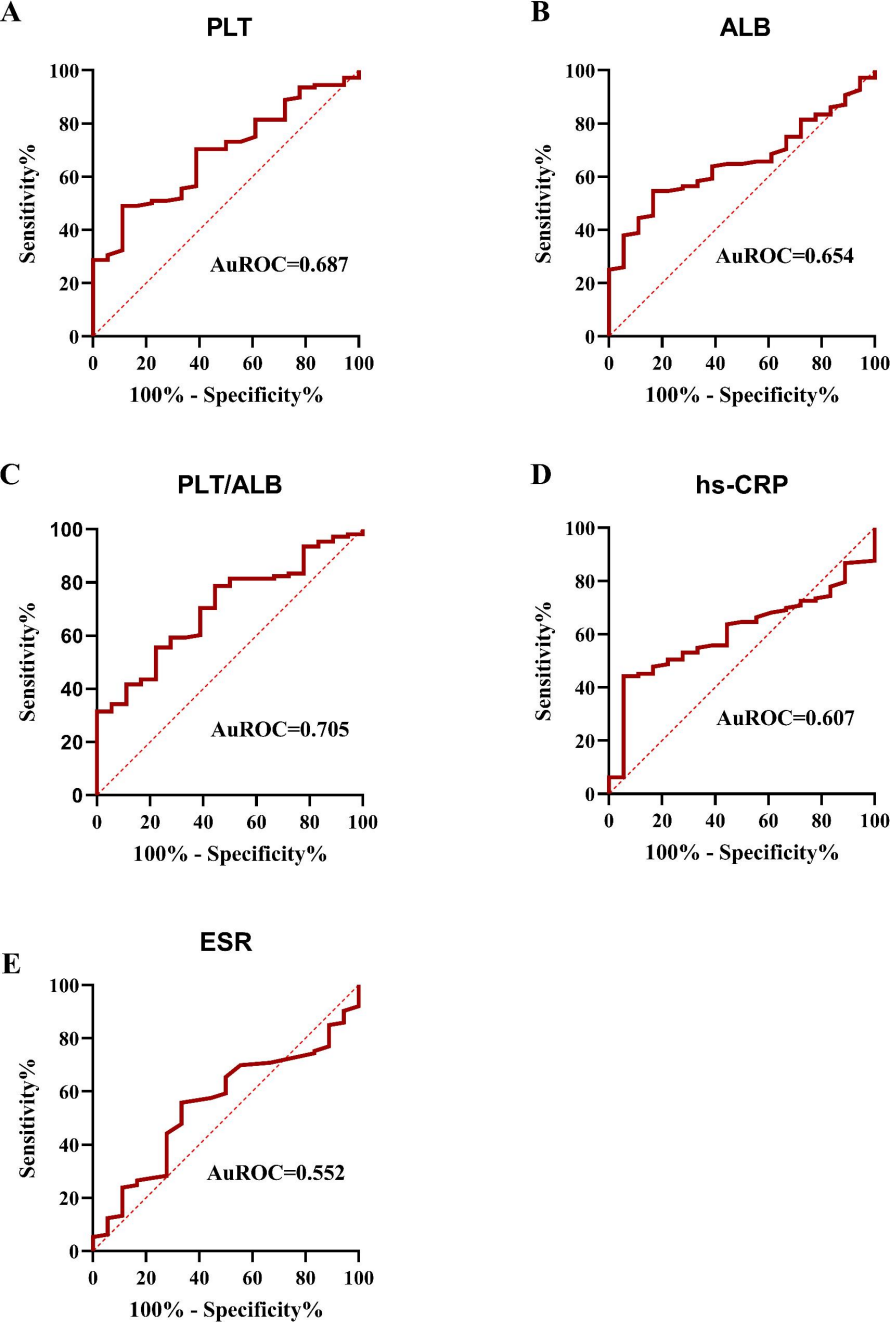
Area under the receiver operating characteristics curve (AuROC) values for (**A**) platelet (PLT), (**B**) Albumin (ALB), (**C**) platelet-to-albumin ratio (PLT/ALB), (**D**) high sensitive C-reactive protein (hs-CRP), and (**E**) erythrocyte sedimentation rate (ESR) levels in patients with ulcerative colitis (UC).

**Fig. 4:**
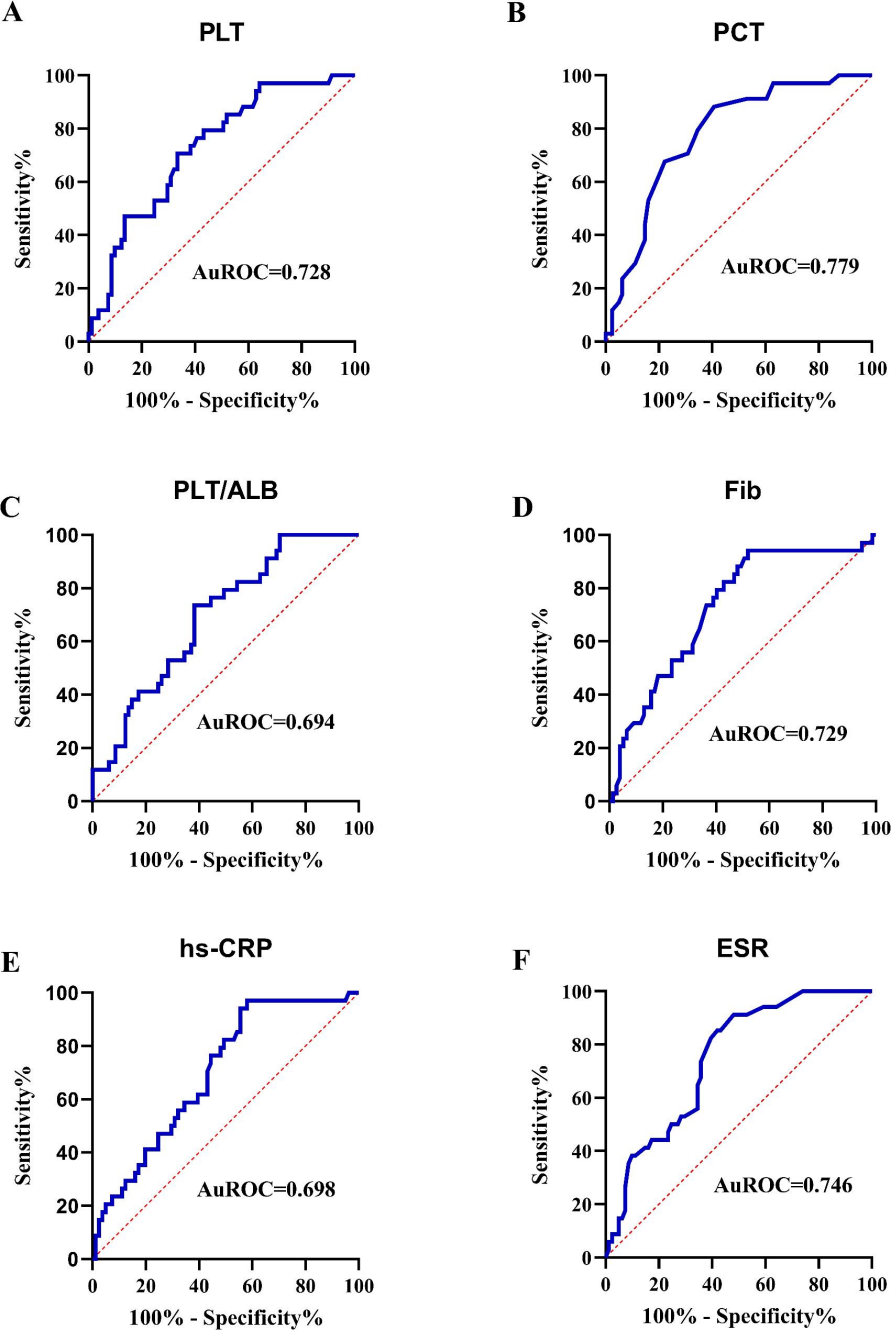
Area under the receiver operating characteristics curve (AuROC) values for (**A**) platelet (PLT), (**B**) plateletcrit (PCT), (**C**) platelet-to-albumin ratio (PLT/ALB), (**D**) Fibrinogen (FIB), (**E**) high sensitive C-reactive protein (hs-CRP), and (**F**) erythrocyte sedimentation rate (ESR) levels in patients with Crohn’s disease (CD).

To evaluate biomarker predictive accuracy with respect to endoscopic disease activity, ROC curves were generated (Figs. 3 and 4). Biomarker AUC values (plus 95% confidence intervals (95% CI)) used to assess endoscopic disease activity in patients with UC and CD are shown (Table 4). The PLT/ALB AUC value for assessing high endoscopic UC activity was 0.705 (95%CI: 0.591–0.819), which was higher when compared with PLT, ALB, ESR, and CRP values in UC patients. The optimal PLT/ALB cut-off value in assessing high endoscopic UC activity was 5.88, with sensitivity and specificity at 0.787 and 0.556, respectively.

**Table 4:** Discriminatory power of each biomarker for endoscopic IBD activity by ROC curves

		AuROC (95% CI)	Cut-off value	Sensitivity	Specificity	Youden’s J statistic
**UC**	**PLT × 10** ^**9**^ **/L**	0.687 (0.573, 0.802)	> 286.50	0.491	0.889	0.380^***^
	**ALB (g/L)**	0.654 (0.547, 0.762)	< 34.50	0.546	0.833	0.400^***^
	**PLT/ALB**	0.7050.591, 0.819)	> 5.880	0.787	0.556	0.343^***^
	**hs-CRP (mg/L)**	0.607 (0.497, 0.719)	> 14.750	0.444	0.944	0.389
	**ESR (mm/hr)**	0.552 (0.428, 0.688)	> 16.50	0.565	0.667	0.231
**CD**	**PLT × 10** ^**9**^ **/L**	0.728 (0.615, 0.814)	> 318.5	0.706	0.649	0.355^***^
	**PCT (%)**	0.779 (0.678, 0.860)	> 0.235	0.882	0.571	0.454^***^
	**PLT/ALB**	0.694 (0.575, 0.781)	> 8.823	0.735	0.597	0.333^***^
	**FIB (g/L)**	0.729 (0.626, 0.826)	> 3.840	0.941	0.481	0.422^***^
	**hs-CRP (mg/L)**	0.698 (0.581, 0.786)	> 7.450	0.910	0.390	0.360^***^
	**ESR (mm/hr)**	0.746 (0.640, 0.828)	> 15.50	0.853	0.558	0.411^***^

*p < 0.05 was considered statistically significant; **p < 0.01; ***p < 0.005

Low endoscopic disease activity: Mayo 0 or 1 in UC and SES-CD < 10 in CD; High endoscopic disease activity: Mayo 2 or 3 in UC and SES-CD ≥ 10 in CD.

**Abbreviations**: ROC, Receiver operating characteristics; hs-CRP, high sensitive C-reactive protein; ESR, erythrocyte sedimentation rate; FIB, fibrinogen; PLT, platelet; PCT, plateletcrit; ALB, Albumin; PLT/ALB, platelet-to-albumin ratio

In patients with CD, PCT, PLT, FIB, and ESR AUC values were > 0.7. PCT had the best discriminative capacity for high endoscopic CD activity. The PCT Area under the receiver operating characteristics curve value was 0.769 (95%CI: 0.678–0.860) and cut-off, sensitivity, and specificity values were 0.235, 0.882, and 0.571, respectively.

## Discussion

In this study, we identified significantly increased PLT/ALB ratio, PLT, PCT, PLR, FIB, hs-CRP, and ESR levels, and significantly decreased ALB and Hb levels in IBD patients when compared with HCs. ROC analyses showed that PLT/ALB ratio (AUC = 0.705), PLT (AUC = 0.687), and ALB (AUC = 0.654) levels were better biomarkers of endoscopic UC activity when compared with routinely applied hs-CRP (AUC = 0.607) and ESR (AUC = 0.552). PCT (AUC = 0.779), ESR (AUC = 0.746), PLT (AUC = 0.728) and FIB (0.729) were better biomarkers of endoscopic disease activity in CD than hs-CRP (AUC = 0.698).

Endoscopic disease activity assessments are essential for diagnosis, prognosis, and evaluating IBD treatment effects [22, 23]. However, due to high costs, invasiveness, and discomfort limitations for patients, concerted research efforts have been made to identify convenient and less expensive biomarkers to evaluate endoscopic IBD activity [21, 24]. Clinical disease activity is subjective and not a reliable indicator of endoscopic IBD activity. Previous studies reported that up to 50% of patients in clinical remission had endoscopic evidence of active IBD, and a high prevalence of patients with obvious clinical symptoms had achieved mucosal healing [25, 26]. Thus, blood-based biomarkers have the potential to function as effective monitoring tools for IBD inflammation and activity.

Currently, serum CRP and ESR levels are routinely used in active IBD [24]. CRP responses are different in UC and CD; the latter disease is significantly correlated with CRP levels but the former is not [27], with unknown underlying mechanisms. ESR reflects red blood cell migration in plasma; during inflammation, ESR time to peak and decline is delayed when compared with CRP [28].

PLTs are involved in active disease periods and not only regulate coagulation but also enhance mucosal inflammation [29]. PLT changes have been described in IBD and include morphological alterations (mean platelet volume (MPV), platelet distribution width (PDW) and PCT) and count increases, which are linked to PLT activation induced by inflammatory agonists [30, 31]. MPV was negatively correlated with some inflammation markers, including CRP and ESR [32], while PCT percentages were markedly correlated with CRP and ESR [33]. ALB synthesis is influenced by inflammatory processes, with levels decreased during inflammatory states [18], however, relationships between PLT/ALB ratios and IBD activity are unclear.

In this study, Mayo scores were positively correlated with increased inflammatory parameters, including hs-CRP, PLT, and PLT/ALB, and negatively correlated with ALB levels in patients with UC. Also, a clear positive correlation was identified between SES-CD scores and several inflammatory biomarkers, including hs-CRP, ESR, WBC, FIB, PLT, PCT, and PLT/ALB in patients with CD. Additionally, our ROC analyses revealed that the optimal PLT/ALB ratio cut-off was 5.88 (sensitivity: 78.7%, specificity: 55.6%, and AUC: 0.705) in UC patients, and the optimal PCT cut-off was 0.235 (sensitivity: 88.2%, specificity: 57.1%, and AUC: 0.779) in CD patients. Interestingly, routinely used biomarkers, including hs-CRP and ESR, had less discriminative values for differentiating between UC patients with either remissive or mild endoscopic UC activity and patients with moderate or severe endoscopic UC activity. ESR, PLT, and FIB also had better discriminative values for CD patients, with AUC values of > 0.7.

In summary, our study found that the AUC for the PLT/ALB ratio was highest in UC, while the AUC for PCT was highest in CD. This suggests that they are optimal for diagnosing endoscopic severity in each respective disease. The PLT/ALB ratio serves as a novel marker for assessing IBD activity. PLT are acute phase reactants that are induced by inflammatory cytokines [34]. Increasing evidence suggests that PLT may also have an impact on fibrosis in gastrointestinal diseases [35]. On the other hand, ALB synthesis is influenced by inflammatory processes, and its levels can reflect the chronicity of severe IBD in terms of bowel damage and mechanical obstruction [36, 37]. In this study, our interest in PLT/Alb was to capture the relative intensity of acute inflammation and chronic bowel damage, even the intestinal fibrosis.

When compared with previous reports, our study had several distinct advantages. Patient numbers were high, which provided a numerical robustness to our study. Furthermore, while endoscopy is widely regarded as a gold standard for evaluating IBD activity, it is expensive, invasive, and difficult and inconvenient for repeated patient examinations. Nevertheless, the biomarkers identified in this study are inexpensive and easily acquired via routine laboratory procedures. Additionally, ours is the first study to evaluate the PLT/ALB biomarker in defining endoscopic IBD activity in patients.

However, our study had some limitations. Firstly, although PLT/ALB ratio and PCT had better discriminative values for endoscopic disease activity, the AUC values of them are only slightly higher than 0.7, which could not be considered as ideal diagnostic biomarkers. Therefore, endoscopic workup remains crucial to determine the progression of IBD. Secondly, we did not establish cause-effect associations between inflammatory biomarkers and IBD activity due to the retrospective nature of our study. Therefore, more prospective studies are required to confirm our results.

## Conclusions

PLT-associated indicators, including PCT and PLT/ALB ratios had the best diagnostic values for endoscopic CD and UC activity, respectively, and were much better than current routine laboratory tests. Importantly, theses biomarkers may improve intestinal inflammation monitoring and therapeutic efficacy in IBD.

## Data Availability

The datasets used and/or analyzed during the current study are available from the corresponding author on reasonable request.

## Abbreviations

ALB: Albumin
AUC: The area under the curve
CD: Crohn’s disease
ESR: Erythrocyte sedimentation rate
FIB: Fibrinogen
Hb: Hemoglobin
HBI: Harvey Bradshaw Index
HCs: Healthy controls
hs-CRP: Highly sensitive C-reactive protein
IBD: Inflammatory bowel disease
IQRs: Interquartile ranges
MPV: Mean platelet volume
NLR: Neutrophil-to-lymphocyte ratios
PCT: Plateletcrit
PDW: Platelet distribution width
PLR: Platelet-to-lymphocyte
PLT: Platelets
PLT/ALB: Platelet-to-albumin ratio
ROC: Receiver operating characteristic
SCCAI: The Simple Clinical Colitis Activity Index
SES-CD: Simple Endoscopic Scores for CD
UC: Ulcerative colitis
WBCs: White blood cells
